# Supplementary material for: Prevalence of pterygium and its associated factors among adults aged 18 years and above in Gambella town, Southwest Ethiopia, May 2019
Source: PLoS One. 2020 Sep 3;15(9):e0237891. doi: 10.1371/journal.pone.0237891 (PMC7470263; doi:10.1371/journal.pone.0237891)
Supplement: S1 Table — (DOCX) [file pone.0237891.s001.docx]

**S1 Table.** Multivariable analysis final out put in a study to assess prevalence of pterygium and its associated factors among adults aged 18 years and above in Gambella town, Southwest Ethiopia. (SAV output)
