# Supplementary material for: Prevalence of pterygium and its associated factors among adults aged 18 years and above in Gambella town, Southwest Ethiopia, May 2019
Source: PLoS One. 2020 Sep 3;15(9):e0237891. doi: 10.1371/journal.pone.0237891 (PMC7470263; doi:10.1371/journal.pone.0237891)
Supplement: S1 File — (DOCX) [file pone.0237891.s002.docx]

**S1 File:** English form of the questionnaire for the study “prevalence of pterygium and its associated factors among adults aged 18 years and above in Gambella town, Southwest Ethiopia, May 2019”.

| 1. **Demographic characteristics** | | | | |
| --- | --- | --- | --- | --- |
| **No** | **Questions** | | **Responses/ answers** | **Remark** |
| **1** | **Age** | | **-------------** | **In years** |
| **2** | **Sex** | | 1. Male 2. Female |  |
| **3** | **Marital status** | | 1. Married 2. Widowed 3. Divorced 4. Single |  |
| **4** | **Educational level** | | 1. Unable to read write 2. Able to read and write 3. Primary school 4. Secondary school 5. College/university |  |
| **5** | **Occupation** | | 1. Student 2. Farmer/labour 3. Employed 4. Merchant 5. Others * | **Employed:** in governmental and non-governmental |
| **6** | **Address** | | 1. Urban 2. Rural |  |
| **7** | **Monthly income** | | ------------------- | **In birr** |
| **8** | **Religion** | | 1. Orthodox 2. Muslims 3. Protestant 4. Catholic 5. Others |  |
| **8** | **Family size** | | 1. 0-3 2. 4-6 3. ≥ 7 |  |
| 1. **Environmental and behavioral factors** | | | | |
| **9** | **Sun exposure** | | 1. Yes 2. No | **If no go to no 11** |
| **10** | **For how much time do you expose to sun light?** | | ------------------ | **In hours** |
| **11** | **Working area** | | 1. Indoor 2. Outdoor | **Outdoor:** Spending 5 hour and above |
| **12** | **Dust exposure** | | 1. Yes 2. No |  |
| **13** | **Sunglass use** | | 1. Yes 2. No | **If no go to no 15** |
| **14** | **How many hours do you wear it per day?** | | 1. Below 5 hours 2. 5 hours and above |  |
| **15** | **Alcohol use** | | 1. Never 2. Past 3. Current |  |
| **16** | **Smoking** | | 1. Never 2. Past 3. Current |  |
| **17** | **History of traditional medication** | | 1. Yes 2. No |  |
| **18** | **Family history of pterygium** | | 1. Yes 2. No |  |
| 1. **Physical examination of the eye (symptoms)** | | | | |
| **19** | 1. Feel of dryness 2. Burning sensation 3. Redness 4. Tearing 5. Visual reduction 6. Irritation 7. Foreign body sensation(grittiness) | | | **Incircle the symptoms** |
| 1. **Physical examination of the eye (symptoms)** | | | | |
| **20** | 1. Redness on the conjunctiva 2. Vascularization (hypermic conjunctiva) 3. Fibrovascular growth on the conjunctiva not reaching at limbus 4. Fibrovascular growth reaching at the limbus 5. Fibrovascular growth reaching at mid iris 6. Fibrovascular growth reaching at pupil margin 7. Fibrovascular growth covering the whole pupil 8. Other signs……… | | | **Incircle all the signs seen** |
| **Assessment of pterygium** | | | | |
| **21** | Presence of pterygium | 1. Yes 2. No | | **If yes go to no 22** |
| **22** | Laterality of pterygium | 1. Unilateral 2. Bilateral | |  |
| **23** | Severity of pterygium | 1. Grade I 2. Grade II 3. Grade III 4. Grade IV | |  |
