# Supplementary material for: Prevalence of pterygium and its associated factors among adults aged 18 years and above in Gambella town, Southwest Ethiopia, May 2019
Source: PLoS One. 2020 Sep 3;15(9):e0237891. doi: 10.1371/journal.pone.0237891 (PMC7470263; doi:10.1371/journal.pone.0237891)
Supplement: Attachment — Submitted filename: Response to Reviwers.docx [file pone.0237891.s004.docx]

**Response to reviewers**

**Manuscript title**: Prevalence of pterygium and its associated factors among adults aged 18 years and above in Gambella town, Southwest Ethiopia, May 2019

**Manuscript number:** PONE-D-20-18073

**Dear reviewers.** Thank you for giving us the chance to revise and correct the manuscript accordingly. We are thankful so much and we appreciate you for careful and in-depth reading of this manuscript and for the thoughtful comments and constructive suggestions, which help us to improve the quality of this manuscript. Saying this we addressed all the concerns raised by you and incorporated our reflection in the revised manuscript. We tried to address all issues that are raised to be addressed.

Many thanks.

**Reviewer reports:**

**Reviewer #1(Catherine McCarty Wallerstein):** As the authors note, there are a number of peer-reviewed publications on the epidemiology of pterygium. Sun exposure is causal. The data in this paper further confirm previous findings of risk factors in a unique population-based study. A few suggestions/questions: 1. How was dust exposure defined?

***Authors’ Response:*** *Thank you! We accepted the comment and we included in the “operational definition” parts of the manuscript.* **Dust exposure:** a person having at least a history of one episode of exposure to dusty environment that causes their eye to be irritated, itchy and red within the past 6 months(1).

**Reviewer #1(Catherine McCarty Wallerstein):** 2. How was sunglass use defined? Did participants have to wear them for a certain percent of tie while outdoors to be considered 'yes'?

***Authors’ Response:*** *Thank you! We accepted the comment and included in the operational definition part of the new revised manuscript.* **Sun glass use was defined as** when participants used any type of glass(cosmetics, general and specially treated) for the purpose of protection from sun during their outdoor activities(2,3).

**Reviewer #1(Catherine McCarty Wallerstein):** 3. Self-reported family history of pterygium is not likely to be accurate and not relevant to this paper, so I suggest deleting.

***Authors’ Response:*** *Thank you! for the comment, the comment is accepted and we deleted it from the new edited manuscript.*

**Reviewer #1(Catherine McCarty Wallerstein):** 4. p<0.25 is an unusual significance level to use. How do the results change is a p<0.10 is used?

***Authors’ Response:*** *Thank you! For the comment.*

commonly used cutting point in bi-variable analysis to multivariable logistic regression is P< 0.20, but some statistician advice that when the variables that enter to multivariable analysis are fewer, it may possible to use the cutting point p<0. 25 and if we tried to use p< 0.10, variables will further be reduced and few not more than five will full fill the inclusion criteria which might reduce the precision of the study rather it is possible to enter all variables. in addition, the criteria developed in cutting the P-value in bivariable analysis is simply to reduce the number of variables that will inter to multivariable analysis. So, it is difficult to use p<.10, in our study since it will affect the whole outcome as well as statistical concept of the study.

**Reviewer #1(Catherine McCarty Wallerstein):** 5. The independent variables in the multivariate analysis are likely highly corrected, this making interpretation challenging. How was that taken into account.

***Authors’ Response:*** *Thank you!* We write in such a way that, in order to be easily understand by the readers and the nature of the variables can not be compressed to a single word easily and we tried to write the manuscript so that the words can easily be understandable.

**Reviewer #1(Catherine McCarty Wallerstein):** 6. If the data are available, it would be interesting to assess a possible dose response relationship between sun exposure and size of pterygium.

***Authors’ Response:*** Thank you! Since sun exposure was operationalized as “those who had exposure for five hours and above are considered as exposed and others as non-exposed” no separate dose of hour was included in the data. So, it is difficult to show the relationship between dose of sun exposure with size of pterygium.

**Reviewer #2(Cristina Schnider):** Thank you for your constructive comment and suggestion, we will try to conduct a future research in the topic/idea you suggested us and we hope we may face you again with that title.

**Responses to accadamic editors**

1. **Response of authors:** *thank you, we tried to correct the manuscript to meet the Plos one’s criteria of manuscript formatting as much as possible.*
2. **Response of authors:** *thank you for your comment, we have already rephrased and correct the duplicated and overlapping texts in the new edited manuscript that were closer to the previous publication.*
3. **Response of authors:** *thank you,*
A. As already mentioned previously, there were no a piece of financial and material support for this study, even the organization that employed us.
B. The funders had no role in the study design, data collection and analysis, decision to publish, or preparation of the manuscript.
C. None of the authors received a salary from funders.
D. The authors received no specific funding for this work.
4. *Thank you!*
A. There is no ethical or legal restriction on sharing the data requested from us
B. Data are uploaded as “supporting information files” at the end of the new edited manuscript.
5. My ORCID iD is already updated from my PLOSE ONE account.
6. The ethics statement is already moved to method part in the new edited manuscript

***References for comments reviewer # 1***

1.	De Smedt SK, Nkurikiye J, Fonteyne YS, Tuft SJ, Gilbert CE, Kestelyn P. Vernal keratoconjunctivitis in school children in rwanda: Clinical presentation, impact on school attendance, and access to medical care. Ophthalmology [Internet]. 2012;119(9):1766–72. Available from: http://dx.doi.org/10.1016/j.ophtha.2012.03.041

2.	Backes C, Religi A, Moccozet L, Behar-Cohen F, Vuilleumier L, Bulliard JL, et al. Sun exposure to the eyes: predicted UV protection effectiveness of various sunglasses. J Expo Sci Environ Epidemiol [Internet]. 2019;29(6):753–64. Available from: http://dx.doi.org/10.1038/s41370-018-0087-0

3.	Chorley AC, Evans BJW, Benwell MJ. Solar eye protection practices of civilian aircrew. Aerosp Med Hum Perform. 2015;86(11):953–61.
